# Gastrointestinal Microbiota and Breast Cancer Chemotherapy Interactions: A Systematic Review

**DOI:** 10.7759/cureus.31648

**Published:** 2022-11-18

**Authors:** Denise Csendes, Sai Dheeraj Gutlapalli, Keerthana Prakash, Kiran Maee Swarnakari, Meena Bai, Mohana Priya Manoharan, Rabab Raja, Aneeque Jamil, Aditya Desai, Darshi M Desai, Safeera Khan

**Affiliations:** 1 Internal Medicine, California Institute of Behavioral Neurosciences & Psychology, Fairfield, USA; 2 Medicine, California Institute of Behavioral Neurosciences & Psychology, Fairfield, USA; 3 Internal Medicine Clinical Research, California Institute of Behavioral Neurosciences & Psychology, Fairfield, USA

**Keywords:** probiotics, microbiota diversity, breast cancer, chemotherapy, gastrointestinal microbiota

## Abstract

Breast cancer is the most common type of cancer in women besides basal cell and squamous cell skin cancer. The current systemic therapy guidelines for this heterogeneous disease are mainly based on the molecular subtypes. However, more research is required to improve rates of therapy resistance and prevent side effects. Previous studies have shown that the human gut microbiota may have an important role in carcinogenesis as well as therapy outcomes, but this factor has not yet been integrated into therapy protocols. This systematic review aims to analyze how response rates and side effect profiles of breast cancer systemic therapies may be affected by the gastrointestinal microbiota. A literature search was performed using multiple databases and keywords related to gastrointestinal microbiota, breast cancer, and anticancer drugs. Studies were excluded if they primarily focused on diseases other than breast cancer. Abstracts, reviews, meta-analyses, and animal experiments were also excluded. After screening, nine studies met all selection criteria and included a total of 566 participants. Most studies described the impact of the gut microbiota on therapy response, but a few additionally discussed chemotherapy side effects, probiotics, or antibiotics. In general, diversity and specific microbiota were linked to chemotherapy response as well as prognosis. Microbiota diversity was also predictive of side effects such as neurological symptoms, weight gain, and constipation. The diversity and composition of gastrointestinal microbiota may serve as biomarkers and provide pathways for the optimization of chemotherapy in breast cancer patients.

## Introduction and background

The American Cancer Society estimates that more than 290,000 new breast cancer cases will be diagnosed in the United States in 2022, and one in eight women will be diagnosed with breast cancer in their lifetime [1]. Breast cancer is a heterogeneous disease that is mainly categorized and systemically treated based on molecular subtypes. Approximately 70% of patients have the estrogen or progesterone receptor positive and human epidermal growth factor 2 (ERBB2, formerly known as HER2) negative subtype [2]. These patients are primarily treated with endocrine therapy, and some may also receive chemotherapy. Patients who have tumors that are positive for ERBB2, accounting for 15% to 20% of breast cancer cases, are treated with a combination of ERBB2-targeted therapy and chemotherapy [2]. Tumors that lack estrogen receptor, progesterone receptor, or ERBB2 molecular markers, referred to as triple-negative, make up 15% of breast cancer cases and are generally treated with chemotherapy [2].

Until recently, chemotherapy was the only accepted treatment of triple-negative metastatic breast cancer without breast cancer gene (BRCA) germline mutations [2,3]. Treatment guidelines have been updated to reflect immunotherapy options, including the immune checkpoint inhibitor pembrolizumab in combination with chemotherapy for triple-negative metastatic breast cancer [3-5]. Novel antibody-drug conjugates targeting ERBB2-low subtypes of advanced breast cancer, such as trastuzumab deruxtecan, have led to longer progression-free survival and overall survival compared to chemotherapy [3,6].

Many studies have also examined the role of gastrointestinal microbiota in carcinogenesis [7,8]. For example, breast cancer patients have lower microbiota alpha-diversity, a lower number of species, and different composition of microbiota compared to healthy controls [7]. Studies have also shown specific changes between the microbiota present in healthy breast tissue and breast cancer, adding yet another dimension to the role of microbiota in carcinogenesis [9,10]. Preclinical models have been used to demonstrate that gut microbiota has the potential to modulate response to anticancer therapies as well as resultant side effects [11,12].

Despite the latest advancements in anticancer therapies, the concern of therapy non-responsiveness still needs to be addressed. It is difficult to predict which patients will respond to therapy, and the factors which lead to therapy response or non-response are not yet fully understood [13]. Furthermore, even the newest therapy combinations may cause 53% to 78% of patients to experience grade three or higher treatment-related adverse events [4-6]. The specific effects therapies may have on the human microbiota and modes of preventing therapy side effects remain elusive. Preclinical and clinical research regarding gut microbiota and anticancer therapies has thus far focused on melanoma, renal cell carcinoma, lung cancer, and colorectal cancer [14-17]. In this systematic review, we examine how gastrointestinal microbiota and anticancer drug interactions affect breast cancer therapy responsiveness and side effects.

## Review

Methods

This study adhered to the Preferred Reporting Items for Systematic Reviews and Meta-Analyses (PRISMA) 2020 statement guideline [18,19].

Search Strategy

A comprehensive literature search was performed using keywords and Medical Subject Headings (MeSH) to identify original research in PubMed, MEDLINE, PubMed Central (PMC), Web of Science, and Science Direct databases (Table 1).

**Table 1:** Summary of the search strategy MeSH, Medical Subject Headings

Search Strategy	Query
Regular Keywords	(gastrointestinal OR gut) AND (microbiome OR microbiota OR dysbiosis) AND ("breast cancer") AND ("anticancer therapy" OR chemotherapy OR immunotherapy)
Medical Subject Headings	("Gastrointestinal Microbiome"[Mesh] OR "Gastrointestinal Microbiome/drug effects"[Mesh] OR "Microbiota"[Mesh]) AND ("Breast Neoplasms"[Mesh] OR "Breast Neoplasms/drug therapy"[Mesh]) AND ("Antineoplastic Agents"[Mesh] OR "Antineoplastic Agents/adverse effects"[Mesh] OR "Antineoplastic Agents/toxicity"[Mesh] OR "Neoadjuvant Therapy"[Mesh] OR "Chemotherapy, Adjuvant"[Mesh] OR "Immunotherapy"[Mesh])

Selection Criteria

Peer-reviewed studies eligible for inclusion reported data regarding gastrointestinal microbiota and anticancer therapy, specifically in female breast cancer patients over 18 years of age. Eligible studies were published in English within the last five years, from May 2017 to May 2022. Abstracts, reviews, meta-analyses, animal studies, editorials, and gray literature were excluded.

Quality Assessment

The included studies were critically appraised using standardized assessment tools. Randomized controlled trials (RCTs) were evaluated using the revised Cochrane risk-of-bias tool [20]. A study was considered to be of acceptable quality if at least three or more domains had a low risk of bias and no domains were categorized as high risk of bias. Observational studies were assessed using the Newcastle-Ottawa Scale (NOS), and studies awarded six or more stars were accepted.

Data Extraction

Extracted data included study design, number of participants, study objective, intervention, comparison groups, diversity results, and outcomes associated with specific bacteria. Data extraction was performed independently by two reviewers, and a third reviewer was consulted in case of discrepancy.

Results

Search Results

**Figure 1:**
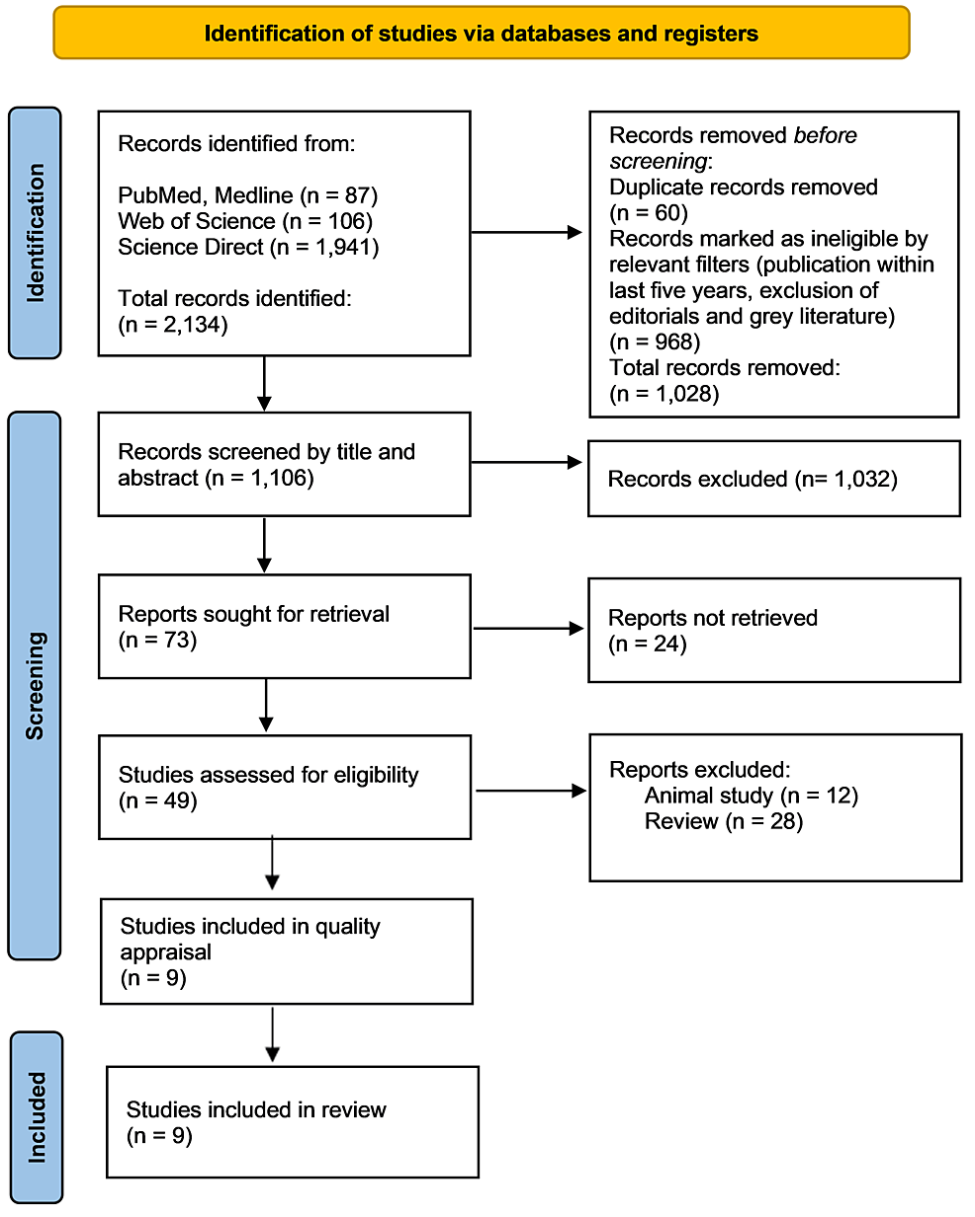
Preferred reporting items for systematic reviews and meta-analyses (PRISMA) 2020 flow diagram

The initial systematic search yielded 2,134 records, of which 60 duplicates were removed. Filters specifying publication dates within the last five years and excluding editorials and grey literature removed another 968 records. The 1,106 remaining articles were screened by title and abstract. Full texts of 49 relevant articles were assessed for eligibility; 40 were excluded due to categorization as animal studies (n = 12) or reviews (n = 28). Nine studies were included in this review, as depicted in the PRISMA 2020 flow diagram (Figure 1) [18].

Study Characteristics

The nine included studies were comprised of three randomized controlled trials and six observational studies. Quality assessments of randomized controlled trials and observational studies are depicted in Tables 2, 3, respectively.

**Table 2:** Summary of revised Cochrane risk-of-bias tool for randomized controlled trials

Selection	Randomization process	Deviations from intended interventions	Missing outcome data	Measurement of the outcome	Selective Reporting	Overall Bias
Juan et al., 2022 [21]	Low	Low	Low	Low	Low	Low
Juan et al., 2021 [22]	Low	Low	Low	Low	Low	Low
Guan et al., 2020 [23]	Low	Some concerns	Some concerns	Low	Low	Some concerns

**Table 3:** Summary of Newcastle-Ottawa Scale for observational studies

Study	Selection				Comparability		Outcome			Score
	Definition of cases	Representative cases	Selection of controls	Definition of controls	Most important factor	Other factors	Ascertainment of exposure	Same methods of ascertainment of both cases and controls	Non-response rate	
Zidi et al., 2021 [24]	*			*	*	*	*	*	*	7
Li et al., 2022 [25]	*	*		*	*	*	*	*	*	8
Terrisse et al., 2021 [26]	*	*	*	*	*	*	*	*		8
Di Modica et al., 2021 [27]	*			*	*	*	*	*	*	7
Uzan-Yulzari et al., 2020 [28]	*	*		*	*	*	*	*		7
Study	Selection				Comparability		Outcome			Score
	Representative exposed cohort	Selection of non-exposed	Ascertainment of exposure	Outcome not present at start	Most important factor	Other factors	Assessment of outcome	Adequate follow-up length	Adequate follow-up	
Zhang et al., 2021 [29]	*	*	*	*	*	*	*	*	*	9

The studies included a total of 566 patients, and individual study populations included up to 159 patients (Table 4). While most studies measured diversity and specific gut microbiota species, additional measured outcomes were incidence of various side effects, disease-free survival, progression-free survival, overall survival, and fecal metabolite profile. The chemotherapy regimens employed varied and included epirubicin, cyclophosphamide, docetaxel, capecitabine, 5-fluorouracil, or trastuzumab.

**Table 4:** Outline of studies analyzing the gut microbiota and breast cancer therapies CRCI, chemotherapy-related cognitive impairment; LDL, low-density lipoprotein; KEGG, Kyoto Encyclopedia of Genes and Genomes; PFS, progression-free survival; DFS, disease-free survival; HER2, human epidermal growth factor 2; OS, overall survival; T3, tumor larger than 5 centimeters; T4, tumor of any size growing into the chest wall or skin, including inflammatory breast cancer

Author and year of publication	Number of patients	Study design	Objective	Key Findings	Conclusion
Juan et al., 2022 [21]	159; probiotics (n=80), placebo (n=79)	Randomized controlled trial	Determine fecal microbiota changes and incidence of cognitive impairment in patients receiving chemotherapy with probiotic supplement or placebo	Patients who received probiotics demonstrated a lower incidence of CRCI (35%) compared to placebo (81%). No difference in alpha-diversity or beta-diversity. Probiotics group had higher proportion of Actinobacteria to Proteobacteria and higher relative abundance of *Enterococcus*. Placebo group had lower relative abundance of *Streptococcus*.	Probiotics helped protect against CRCI and dysbiosis induced by chemotherapy.
Juan et al., 2021 [22]	92; probiotics (n=47), placebo (n=45)	Randomized controlled trial	Determine weight, body fat, and microbiome changes between patients receiving chemotherapy with probiotic supplement or placebo	Patients who received probiotics showed smaller changes in weight (Mean 0.77 vs. 2.70), body-fat percentage (Mean 0.04 vs. 3.86), and LDL (Mean -0.05 vs 0.39). No difference in alpha-diversity or beta-diversity. Probiotics group had increased relative abundance of Tenericutes and *Eubacterium coprostanoligenes*. Placebo group had increased relative abundance of *Bacteroides* and *Anaerostipes*.	Probiotics reduced chemotherapy side effects of dysbiosis, weight gain, and other metabolic changes.
Guan et al., 2020 [23]	31; metronomic capecitabine (n=15), conventional dose capecitabine (n=16)	Randomized controlled trial	Compare the gut microbiome of patients treated with metronomic chemotherapy vs. conventional dose	No statistical difference in alpha-diversity. Beta-diversity of metronomic group lower than conventional dose group. Nine KEGG modules enriched in metronomic group. Presence of *Slackia* associated with decreased PFS (9.2 vs 32.7 months). Patients with *Blautia* had longer PFS (32.7 vs. 12.9 months).	The dose regimen of chemotherapy made a difference in the gut microbiota profile. Certain bacterial species may be utilized as predictive biomarkers.
Li et al., 2022 [25]	23; complete or partial response (n=18), nonresponse (n=5)	Observational	Examine the role of gut microbiota in neoadjuvant chemotherapy responsiveness	Gut microbiota of nonresponsive patients were characterized by low diversity, increased abundance of *Bacteroides*, and decreased *Firmicutes*. *Coprococcus*, *Dorea*, and uncultured *Ruminococcus* species combination differentiated the two groups the best.	Gut microbiota composition differed between responders and patients resistant to treatment.
Zhang et al., 2021 [29]	120; antibiotic-treated (n=55), control (n=65)	Observational	Evaluate effect of antibiotics on neoadjuvant chemotherapy efficacy	The control group exhibited higher pathological complete response rate (29.09% vs. 10.20%). Treatment with antibiotics was associated with reduced DFS. The effect of reduced chemotherapy efficiency was prominent in the HER2-positive group for DFS and OS as well as in the T3-T4 group for DFS and OS.	Antibiotics were associated with reduced chemotherapy efficacy and worse prognosis.
Zidi et al., 2021 [24]	8; good-responders (n=6), nonresponders (n=2)	Observational	Describe microbiota-derived fecal metabolites during neoadjuvant chemotherapy	Chemotherapy affected the gut metabolome after the second treatment cycle. Nonresponders had increased uracil, tyrosine, and acetone. Nonresponders also had significant decreases in butyrate, methanol, and 3-methylhistidine.	Chemotherapy modulated fecal metabolites.
Terrisse et al., 2021 [26]	76	Observational	Evaluate gut microbiota and plasma metabolomics associations with breast cancer prognosis and chemotherapy side effects	Beta-diversity separated patients based on tumor grade and staging. After therapy, alpha-diversity increased. Bacteria found in samples from patients with more aggressive tumors included *Bacteroides*. Bacteria associated with better prognosis included *Coprococcus* genus and *Ruminococcaceae*. Chemotherapy increased the species usually found in healthy volunteers and reduced the species associated with unfavorable prognosis.	Specific microbiota were associated with good prognosis, while other microbiota were associated with poor prognosis. Chemotherapy changed the relative abundance of favorable and unfavorable species.
Di Modica et al., 2021 [27]	24; pathologic complete response (n=16), nonresponsive (n=8)	Observational	Analyze the influence of gut microbiota on the efficacy of trastuzumab-containing neoadjuvant chemotherapy	Patients with complete pathologic response exhibited higher alpha-diversity. Beta-diversity separated patients based on response to treatment. Nonresponsive group had more *Bacteroides*.	Gut microbiota directly impacted trastuzumab efficacy.
Uzan-Yulzari et al., 2020 [28]	33; breast cancer (n=28)	Observational	Investigate whether women who gain weight following chemotherapy have a microbiome that promotes obesity	Significant difference in pretreatment beta-diversity between women who gained weight and those who did not. The patients who gained weight had gut microbiota with higher alpha-diversity and higher relative abundance of Erysipelotrichaceae.	Gut microbiota composition and diversity were associated with weight gain after chemotherapy

Discussion

In this systematic review, we analyzed the most recent evidence of interactions between gut microbiota and anticancer therapies in breast cancer patients, taking into account the varied scope of studies. Five studies described either the effect of gut microbiota on therapy response or the impact of chemotherapy on the gut microbiota. The influence of microbiota on therapy side effects was examined by two studies, and three studies additionally investigated the modulation of therapy by probiotics or antibiotics.

Influence of Gut Microbiota on Chemotherapy Response

Microbiota diversity trends were generally linked to patients’ response to chemotherapy and prognosis. Specific gastrointestinal microbiota species also acted as potential biomarkers predictive of therapy response. Conversely, chemotherapy itself could alter microbiota either towards an advantageous or disadvantageous profile.

In a study conducted by Di Modica et al., patients with pathologic complete response to trastuzumab-containing neoadjuvant chemotherapy exhibited higher pretreatment microbiota alpha diversity than nonresponsive patients [27]. The beta diversity, or the general microbiota composition variability between samples, was significantly different between patients based on their response to treatment [25,27]. The gut microbiota of responders was abundant in Clostridiales, *Bifidobacteriaceae*, *Turicibacteraceae*, and *Prevotellaceae*. Nonresponsive patients had a high abundance of *Bacteroides* and low alpha diversity. Fecal microbiota transplantation to breast cancer model mice replicated the chemotherapy response seen in patients, further supporting the results.

Using comparable patient population size, Li et al. also reported higher pretreatment microbiota alpha-diversity for patients responsive to neoadjuvant chemotherapy, and beta-diversity was significantly different between responsive and nonresponsive patients [25]. Similar to Di Modica et al., an increased abundance of *Bacteroides* was implicated in nonresponse. In addition, the combination of *Coprococcus*, *Dorea*, and uncultured *Ruminococcus* species was relatively decreased in nonresponders.

A slightly larger cohort study, consisting of a subset of 76 patients from the Cancer Toxicity trial (CANTO, NCT01993498), evaluated differences in microbiota between patients with favorable or unfavorable prognoses [26,30]. The study results demonstrated that beta-diversity could stratify patients based on tumor size, grade, axillary lymph node metastasis, and TNM staging [26]. Consistently with other studies, *Bacteroides* was generally associated with a worse prognosis, and *Coprococcus* as well as *Ruminococcaceae* were associated with a more favorable prognosis. Other bacteria found in samples from patients with more aggressive tumors included *Streptococcus*, *Lachnospiraceae*, *Veillonella*, *Enterobacteriaceae*, and *Clostridiaceae* family members. Additional bacteria associated with a better prognosis included *Methanobrevibacter smithii*, *Eubacteriaceae*, *Akkermansia muciniphila*, *Desulfovibrio piger*, *Collinsella*, and *Bacteroides vulgatus*. In a limited subset of patients who underwent neoadjuvant chemotherapy, pathologic complete response was associated with *Gemella sanguinis*, *Streptococcus mitis*, and *Eubacterium ventriosum*. The effects of chemotherapy lead to increased microbiota diversity and a reduction in the abundance of species associated with unfavorable prognoses [26].

A randomized controlled trial of 31 patients comparing metronomic capecitabine to conventional dose showed that the dose of chemotherapy altered the microbiota profile as well [23]. Metronomic dose chemotherapy made no statistical difference in survival or alpha diversity, but it was associated with lower beta diversity. Potential predictive biomarkers identified were *Blautia*, corresponding to increased progression-free survival, and *Slackia*, associated with decreased progression-free survival. A unique feature of this study was the analysis of gut microbiota function via the Kyoto Encyclopedia of Genes and Genomes (KEGG), and several modules were enriched in patients who received metronomic chemotherapy.

Zidi et al. reported that chemotherapy significantly affected the gut metabolic profile after the second cycle, and short-chain fatty acids were increased after chemotherapy [24]. Specific amino acids increased in patients who responded to chemotherapy, but other distinct changes characterized patients who did not respond. In patients who were resistant to chemotherapy, uracil, tyrosine, and acetone increased, while butyrate, methanol, and 3-methylhistidine levels decreased. This observational study, however, was limited by the small patient population size.

Microbiota and Therapy Side Effects

In addition to its role in shaping chemotherapy efficacy, gastrointestinal microbiota may also regulate the incidence of treatment side effects. Terrisse et al. analyzed associations between the fecal microbiota profile and various side effects of therapy [26]. Following chemotherapy, beta diversity was predictive of neurological side effects, weight gain, constipation, or hot flashes. The *Clostridiaceae* family was particularly associated with neurological side effects. A few of the bacteria implicated in the development of neurological symptoms and concurrently associated with poor prognosis were *Enterocloster bolteae*, *Clostridium spiroforme*, *Bacteroides uniformis*, *B. thetaiotaomicron*, and *Erysipelatoclostridium ramosum*. Microbiota associated with poor prognosis were also increased in patients who did not experience weight gain as a side effect.

The results presented by Terrisse et al. simultaneously supported and contradicted those of a previous, smaller study. Uzan-Yulzari et al. reported that the beta diversity before treatment was predictive of weight gain following chemotherapy, but patients who gained weight also had higher microbiota alpha-diversity [28]. In contrast to Terrisse et al., a higher relative abundance of *Erysipelotrichaceae* family members was found in patients who had at least a 3% increase in body weight. Results were further supported by fecal microbiota transplantation in mice. However, the interpretation of the results was inherently limited by the size of the cohort and the inclusion of patients with gynecological malignancies.

Modulation by Probiotics and Antibiotics

Juan et al. conducted two randomized controlled trials which aimed to determine differences in fecal microbiota and incidence of side effects between patients who received a probiotic supplement or placebo during chemotherapy [21,22]. Both studies reported no difference in microbial diversity between the two groups. The first study revealed that probiotic administration during docetaxel-based chemotherapy was associated with stable weight, body fat, glucose, total cholesterol, and low-density lipoprotein (LDL) [22]. The probiotics group had a lower incidence of constipation as well as a higher abundance of Tenericutes and *Eubacterium coprostanoligenes*. The abundance of *Bacteroides*, which was generally associated with unfavorable outcomes in other studies, and *Anaerostipes* were significantly decreased in the probiotics group compared to the placebo. Probiotic supplementation was overall associated with diminished side effects of chemotherapy.

In a second randomized controlled trial by Juan et al., patients who took supplemental probiotics during chemotherapy had a lower incidence of cognitive impairment, constipation, and emesis compared to placebo [21]. Using a different method of analysis and a larger patient population compared to their previous study, the results of Juan et al. once again demonstrated that a probiotic supplement helped maintain glucose and LDL levels during chemotherapy. In accordance with the composition of the probiotic supplement, a subset of the intervention group had abundant *Enterococcus* and an increased proportion of Actinobacteria to Proteobacteria. Changes in *Enterococcus* abundance were connected to changes in the plasma metabolite p-Mentha-1,8-dien-7-ol, which were negatively correlated with chemotherapy-related cognitive impairment. Thus, specific metabolites associated with gut microbiota were proposed to be a part of the mechanism by which probiotics prevent chemotherapy-induced cognitive impairment.

Certain circumstances during chemotherapy, such as febrile neutropenia, necessitate the administration of antibiotics [29]. However, antibiotics may cause gastrointestinal dysbiosis, which has the potential to affect cancer treatment outcomes. An observational study of 120 patients receiving neoadjuvant chemotherapy found that patients who did not receive antibiotics during treatment had higher pathologic complete response rates [29]. Administration of antibiotics was associated with reduced disease-free survival, and it was a prognostic factor for disease-free survival as well as overall survival. The reduction of chemotherapy efficacy was most prominent in HER2 positive and T3-T4 subgroups. Thus, the management of patients undergoing chemotherapy should aim to limit side effects requiring antibiotics as much as possible.

Limitations

The limitations of this study must be taken into account when evaluating the potential for cancer therapy advancements. Currently, few randomized controlled trials evaluate the influence of gut microbiota in breast cancer patients, and this may raise concerns regarding the quality of evidence available. One of the observational studies also included five patients with gynecological malignancies, which was an inconsistency in the homogeneity of patient cohorts [28]. More specific, high-quality evidence from studies published over five years ago or studies published in languages other than English may have been excluded from this systematic review. Finally, the included studies were highly variable in their designs, chemotherapy protocols, primary outcomes, secondary outcomes, and methods of outcome measurement.

## Conclusions

Studies concerning chemotherapy and the gastrointestinal microbiota demonstrated complex interconnections, some of which considerably impacted patient outcomes. In general, microbiota diversity and specific combinations of bacteria may be used to predict which patients are likely to have good prognoses and responses to chemotherapy. Microbiota diversity could also be used to predict the likelihood of developing chemotherapy side effects. In high-risk patients, the addition of a probiotic supplement may help mitigate anticipated side effects. The gut microbiome is rich with untapped potential for advancing personalized medicine.

Despite the limitations of this study, the aforementioned findings may open new avenues for distinct predictive biomarkers and optimization of chemotherapy protocols for breast cancer patients. Harnessing the human microbiota can be a useful component in maximizing the efficacy of chemotherapy and concurrently minimizing overtreatment as well as side effects. Since few bacteria are presently known to have similar effects across multiple studies, larger studies are required to pinpoint relatively consistent combinations of microbiota. Further research is also needed to determine optimal strategies for manipulating specific microbiota abundance safely, either through probiotic supplements, dietary prebiotics, fecal microbiota transfer, or other innovative techniques. The efficacy, risks, and long-term effects of any such modifications should also be critically assessed.
